# Recombinant IFN-γ from the bank vole *Myodes glareolus*: a novel tool for research on rodent reservoirs of zoonotic pathogens

**DOI:** 10.1038/s41598-018-21143-0

**Published:** 2018-02-12

**Authors:** Francesca Torelli, Steffen Zander, Heinz Ellerbrok, Georg Kochs, Rainer G. Ulrich, Christian Klotz, Frank Seeber

**Affiliations:** 1 Department of Mycotic and Parasitic Agents and Mycobacteria, Robert Koch-Institut, Berlin, Germany; 2 Center for Biological Threats and Special Pathogens, Highly Pathogenic Viruses, Robert Koch-Institut, Berlin, Germany; 3 Institute of Virology, Medical Center – University of Freiburg, Faculty of Medicine, University of Freiburg, Freiburg, Germany; 4 Institute of Novel and Emerging Infectious Diseases, Friedrich-Loeffler-Institut, Federal Research Institute for Animal Health, Greifswald-Insel Riems, Germany

## Abstract

Rodent species like *Myodes glareolus* and *Microtus* spp. are natural reservoirs for many zoonotic pathogens causing human diseases and are gaining increasing interest in the field of eco-immunology as candidate animal models. Despite their importance the lack of immunological reagents has hampered research in these animal species. Here we report the recombinant production and functional characterization of IFN-γ, a central mediator of host’s innate and adaptive immune responses, from the bank vole *M*. *glareolus*. Soluble dimeric rec*Mg*IFN-γ was purified in high yield from *Escherichia coli*. Its activity on *M*. *glareolus* and *Microtus arvalis* kidney cell lines was assessed by immunofluorescent detection of nuclear translocation and phosphorylation of the transcription factor STAT1. Rec*Mg*IFN-γ also induced expression of an IFN-γ-regulated innate immunity gene. Inhibition of vesicular stomatitis virus replication in vole cells upon recMgIFN-γ treatment provided further evidence of its biological activity. Finally, we established a rec*Mg*IFN-γ-responsive bank vole reporter cell line that allows the sensitive titration of the cytokine activity via a bioluminescence reporter assay. Taken together, we report valuable tools for future investigations on the immune response against zoonotic pathogens in their natural animal hosts, which might foster the development of novel animal models.

## Introduction

Rodents are a major reservoir of zoonotic pathogens, which cause nearly 60% of human infectious diseases^1^. These infectious agents pose a public health threat to humans and livestock alike^2^. Besides the widely studied house mouse *Mus musculus* and the Norway rat *Rattus norvegicus* other rodent species have a crucial role as carriers of pathogens in the wild. Within the same family Muridae this includes the wood mouse *A*. *sylvaticus* and within the family Cricetidae the deer mouse *Peromyscus maniculatus*, the bank vole *Myodes glareolus* and the common vole *Microtus arvalis* (called “non-murine rodents” further on). For named species genome sequences are available, thus allowing detailed molecular and immunological studies. The importance of voles as host species for pathogens is emphasised by our own analysis of data from a recent report showing that out of 217 rodent species known to be reservoirs for zoonotic pathogens, 43 (19.8%) belong to these four genera^3^ (Supplementary Fig. S1). Furthermore, in the respective study, modelling suggested that out of 57 rodent species predicted to be reservoirs for future zoonoses, 16 (28%) are vole species.

Of these rodent species the bank vole has attracted increased attention for being the main host for Puumala orthohantavirus (PUUV), one of the most important hantaviruses in Europe. In these voles infection with PUUV is thought to be almost asymptomatic whereas it usually provokes a mild to moderate form of hemorrhagic fever with renal syndrome (HFRS) in humans, occasionally with fatal outcome^4^. Recent studies suggest that the bank vole is a reservoir not only for tick-borne encephalitis virus but also for cowpox virus, a pathogen increasingly reported to cause infection in humans^5–7^. In addition, bacteria such as *Leptospira* spp., *Bartonella* spp., *Rickettsia* spp., *Borrelia* spp. and unicellular eukaryotic parasites like *Giardia* spp., *Cryptosporidium* spp., *Babesia microti* and *Toxoplasma gondii* are just some of the pathogens identified in non-murine rodents^8–13^. Thus, voles and other rodents are important carriers of pathogens but are largely understudied.

Non-murine rodents have also found increased interest in the field of wild- or eco-immunology. The laboratory *M*. *musculus* system is often a choice based on convenience rather than closeness to the natural situation and its value has been largely questioned, especially in the context of translational research^14^. Therefore, non-murine rodent models are gaining increased attention since they are natural reservoirs of many zoonotic pathogens and carriers of the genetic variability lost in inbred mouse colonies^15,16^. The lack of crucial reagents like species-specific cytokines greatly hampers their more widespread use, but recent availability of genomic and transcriptomic data^17,18^ make immunological studies feasible also for non-murine rodents^19^.

Interferon γ (IFN-γ) is one of the main cytokines regulating immunity and inflammation. Its eminent role in Th1 immune responses, macrophage activation, autoimmunity, host defense against intracellular pathogens and modulation of the adaptive immunity is well understood^20^. IFN-γ binds as a homodimer to its cell surface receptor –composed of subunits IFN- γR1 and IFN- γR2– leading to subsequent activation of the Janus Kinase (JAK)-mediated signal transduction pathway^21^. Signal transducer and activator of transcription 1 (STAT1) is the main transcription factor that is activated by engagement of the IFN-γ receptor. Phosphorylation of tyrosine 701 (Tyr701) and subsequent dimerization of STAT1 leads to its nuclear translocation where it can induce the expression of several target genes^22^ via binding to the interferon-Gamma Activator Sequence (GAS) in their promoter region. For its maximal transcriptional activity, phosphorylation of serine 727 (Ser727), mediated by the Mitogen-Activated Protein (MAP) kinase pathway, is also needed^23^. In mice, the family of Immunity Related GTPase (*IRG*) genes is among the many induced by IFN-γ^24^. The corresponding large group of IRG proteins regulates cell-autonomous antimicrobial responses of the host following infection by intracellular pathogens like the bacterium *Chlamydia trachomatis* or the protozoan *T*. *gondii*^25,26^. IFN-γ is also a major mediator of the immune response against many viruses, including vesicular stomatitis virus (VSV)^27,28^.

While signalling via the IFN-γ pathway is conserved among different organisms, the cytokine and its receptors are species-specific and show no or only little cross-reactivity^29^. Consequently, in most cases the homologous protein is needed when experimentally working with a given vertebrate species. For less studied species commercial IFN-γ sources are scarce or not available and in all those cases cloning of its coding sequence and production of the protein is needed for experimental work. Here we report the generation and functional characterization of recombinant IFN-γ of the bank vole *M*. *glareolus*.

## Results and Discussion

### Limited sequence conservation of IFN-γ between different rodent species and its implication for activity

**Figure 1:**
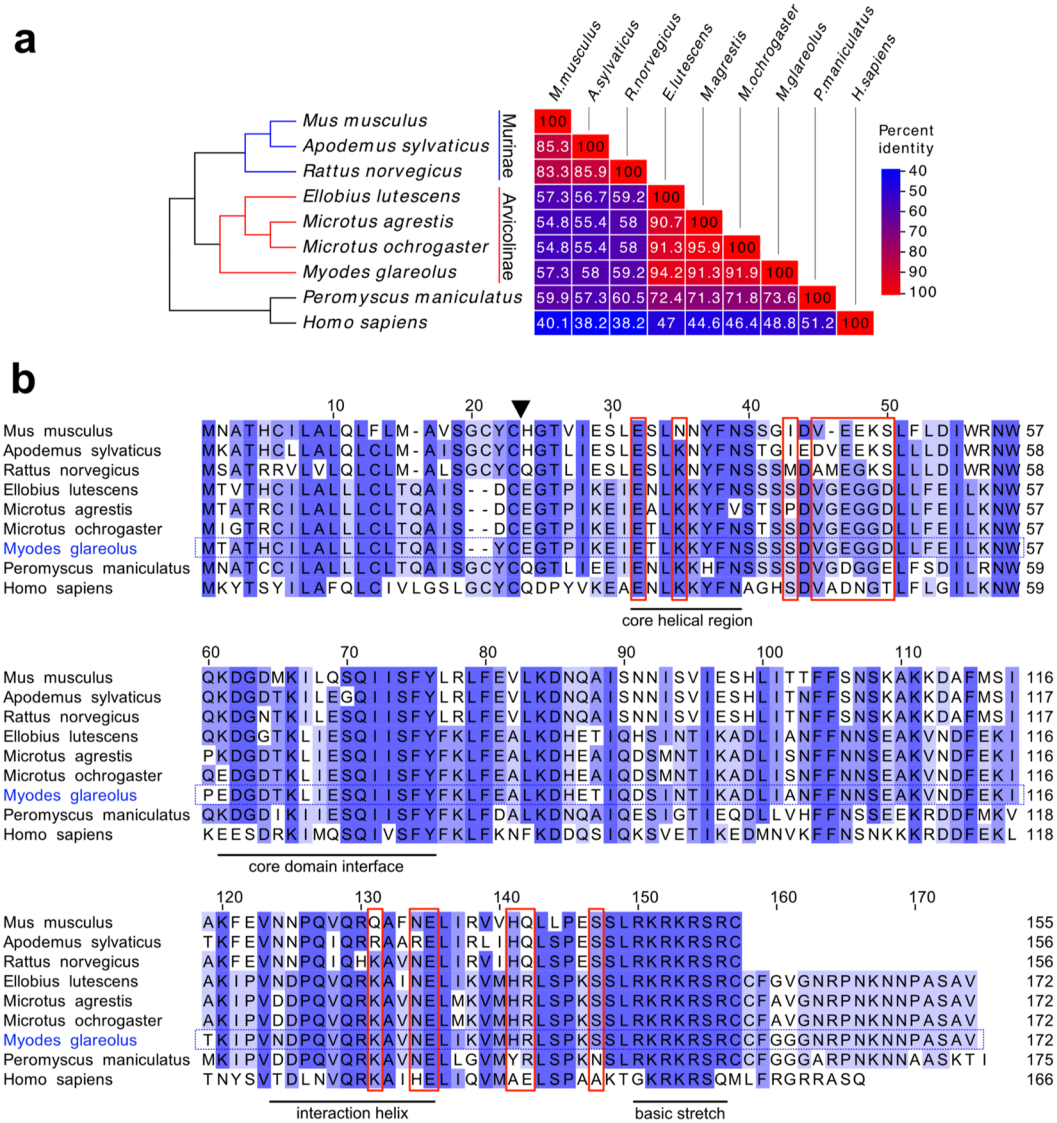
Protein sequence diversity of IFN-γ within rodent species. (**a**) Amino acid sequence similarity (in percentage) of IFN-γ proteins. On the left, a taxonomic species tree is shown, whereby *Peromyscus maniculatus* and *H*. *sapiens* served as more distant species for comparison. The Arvicolinae (red branches) and the Murinae species (blue branches) are indicated. (**b**) IFN-γ amino acid sequence comparison between different rodent species and *H*. *sapiens*. Light blue indicates lower percentage of identity than dark blue. Arrow head indicates the *M*. *musculus* signal peptide cleavage site. Residues involved in the binding to the receptor subunit IFN-γR1 are highlighted by red boxes. Functionally important regions and the C-terminal basic domain are indicated^29^.

The known species-specific activity of IFN-γ is reflected by diversity at the protein sequence level^29^. We compared available IFN-γ genomic sequences of various rodents with that of *Homo sapiens* for comparison (Fig. 1a). The cluster with the highest degree of amino acid sequence conservation is represented by the arvicoline species *Microtus ochrogaster*, *M*. *agrestis*, *Ellobius lutescens* and *M*. *glareolus*, with more than 90% sequence identity over the entire sequence, followed by the murine cluster with *M*. *musculus*, *A*. *sylvaticus* and *R*. *norvegicus* (Fig. 1a). In contrast there is a high divergence between murid and arvicoline clusters, with *M*. *musculus* and *M*. *glareolus* sharing only 57.3% amino acid sequence identity.

At the protein level high sequence conservation of IFN-y exists between species in streches corresponding to core regions of the protein (underlined areas in Fig. 1b) whereas it is lower outside of the same phylogenetic clade. In particular, certain residues previously implicated in the binding of IFN-γ with the receptor (IFN-γR1)^29^ have a high degree of sequence divergence between murid and cricetid rodents (red boxes in Fig. 1b).

A further obvious difference is the C-terminal extension of the IFN-γ sequence of cricetid rodents as well as of humans compared to that of murid rodents (Fig. 1b). The basic lysine and arginine-rich stretch, located at the carboxy-terminal end of murine IFN-γ (Fig. 1b; “basic stretch”), is highly conserved in all IFN-γ sequences and has also been implicated in receptor binding^29,30^. It is interesting to note that carboxy-terminal deletion mutants (up to 10 aa) of recombinant human IFN-γ up to the basic stretch showed higher antiviral activity than the corresponding wild-type cytokine^31–34^. This enhanced activity was attributed to a higher thermal stability of the truncated protein, measured by circular dichroism analysis^33^. It is tempting to speculate that the extension beyond the basic stretch has evolved differently in different species to allow fine-tuning of the interaction between IFN-γ and its specific receptor molecules and thus cellular activation.

Taken together, there is low sequence conservation between murid and cricetid sequences and we anticipated and confirmed (see below) that commercial mouse IFN-γ would be active on mouse and murid cells but not on cells of vole species. Therefore, heterologous expression of *M*. *glareolus* recombinant IFN-γ (rec*Mg*IFN-γ) was attempted in *Escherichia coli*.

### Production of dimeric recombinant *Mg*IFN-γ in *E*. *coli*

**Figure 2:**
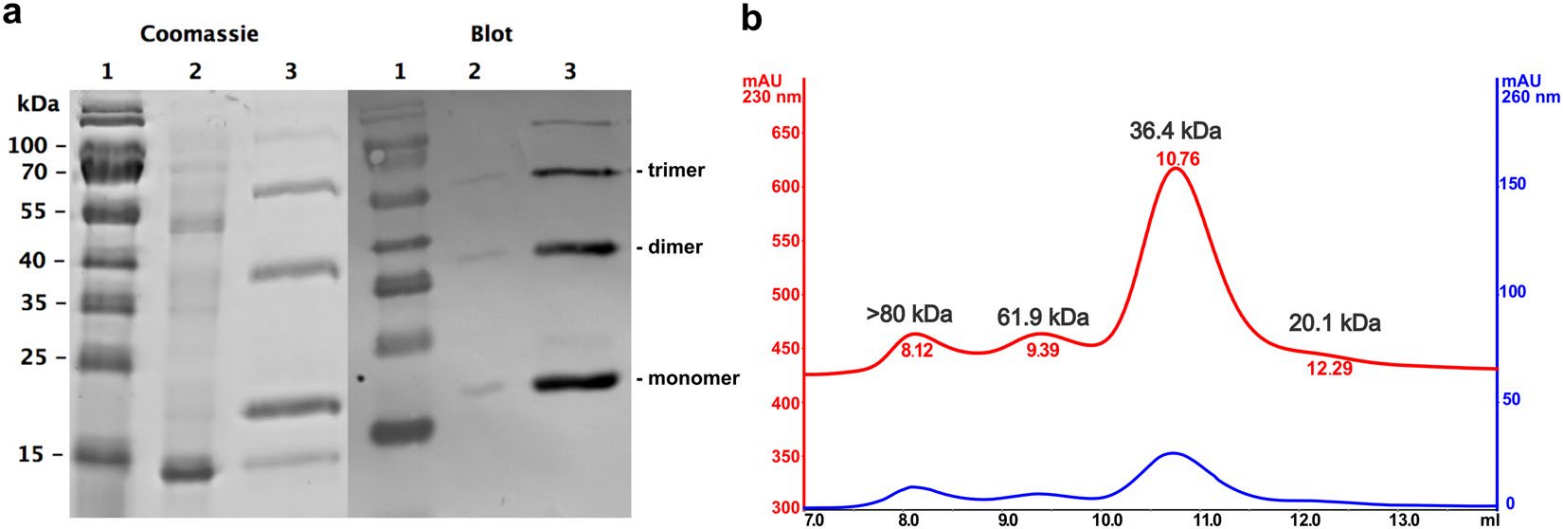
rec*Mg*IFN-γ purified by immobilised metal affinity chromatograph is mostly in a dimeric form. (**a**) SDS-PAGE followed by Coomassie staining for total protein detection (left) and detection of 6His-tagged proteins by Western Blot with an anti-6His monoclonal antibody (right). Lane 1: molecular weight marker (with sizes given to the left - sizes greater than 100 kDa were unreliable when constructing a standard curve); lane 2: flow-through (unbound proteins); lane 3: purified protein(s). (**b**) Size determination of purified rec*Mg*IFN-γ by analytical gel filtration analysis on a pre-calibrated Superdex 75 column. Protein peaks were detected at 230 nm (red) and 260 nm (blue), respectively.

Based on a deposited *Mg*IFN-γ sequence (GenBank HQ650825) an *E*. *coli* expression vector was designed that allowed the recombinant expression of *Mg*IFN-γ as a C-terminally 6His-tagged protein (rec*Mg*IFN-γ). Expression was robust, yielding generally > 3 mg/L of soluble protein after a single round of purification by immobilised metal affinity chromatography. SDS-PAGE analysis of the purified protein showed three major bands (20, 42 and 65 kDa; inferred from a standard curve based on the MW markers) and two minor bands (15 and > 100 kDa; Fig. 2a). The four largest proteins reacted with an anti-6His antibody and are interpreted as monomer, dimer, trimer and higher order aggregates. Such aggregates are known to occur with recIFN-γ under non-native conditions^35–38^, as is e.g. the case during SDS-PAGE. Their molecular masses are in good agreement with the calculated MW of monomeric rec*Mg*IFN-γ containing a 6His-tag (18.3 kDa).

The bacterial strain OmniMax 2T1R (containing the outer membrane protease OmpT) provided better expression results than the OmpT-deficient strain BL21 in our hands. However, OmpT is known to cut within the C-terminal stretch of basic amino acids of *H*. *sapiens* IFN-γ^39,40^ resulting in the frequently observed truncated, but otherwise stable ca. 15–16 kDa version of *E*. *coli*-derived IFN-γ^33,39^. In different preparations the small 15 kDa protein not reactive with an anti-6His antibody was inconsistently seen. We therefore assume that this protein is a C-terminal degradation product and is co-purified by dimer formation with a 6His-tagged monomer.

Since IFN-γ needs to be in a homodimeric form to activate its receptor we analyzed purified rec*Mg*IFN-γ by gel filtration on a calibrated analytical Superdex 75 column to determine the proportion of the dimeric form in our preparations. As shown in Fig. 2b, the vast majority of the applied protein eluted at a column volume which is consistent with the dimeric form (36.4 kDa observed vs. 36.6 kDa calculated). The peaks at around 20 kDa and 62 kDa presumably represent monomeric and trimeric forms, and the peak >80 kDa higher order aggregates, respectively (column resolution is up to 70–80 kDa). Collectively, these results show that a single affinity chromatography purification of rec*Mg*IFN-γ resulted in a large proportion of soluble, dimeric and thus presumably correctly folded and active protein.

### rec*Mg*IFN-γ activates STAT1 signaling in vole cell lines

When IFN-γ activates its signaling pathway phosphorylation of cytosolic STAT1 and its subsequent nuclear translocation is observed^22^. This signalling cascade and the molecules involved are highly conserved between species and present even in fish^41^. We therefore assessed co-localization of phospho-Tyr701-STAT1 with cell nuclei of treated cells by immunofluorescent assay (IFA) with a rabbit anti-phospho Tyr701-STAT1 antibody (similar to a previous study on bank voles^42^) on different cell lines (Table 1).

**Table 1:** Rodent cell lines used in the present work.

Name of cell line	Species	Common name	Organ/Tissue	Cell type
NIH/3T3	*Mus musculus*	House mouse	Embryo	Fibroblast
BVK168	*Myodes glareolus*	Bank vole	Kidney	Epithelial-like
FMN-R	*Microtus arvalis*	Common vole	Kidney	Epithelial-like
AAL-R	*Apodemus agrarius*	Striped field mouse	Lung	Epithelial and fibroblast-like

**Figure 3:**
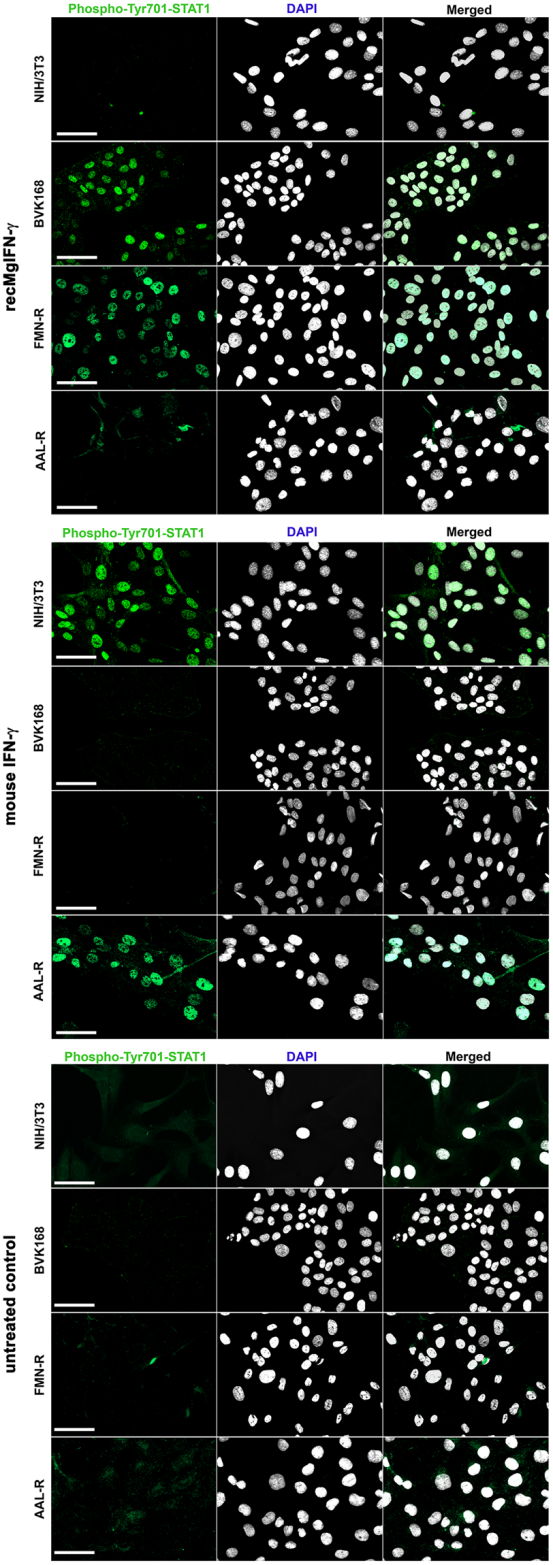
Species-specific activity of rec*Mg*IFN-γ and mouse IFN-γ on different rodent cell lines. NIH/3T3, BVK168, FMN-R and AAL-R cells were tested and either left untreated as control (lower panel), stimulated for 1 h with rec*Mg*IFN-γ (200 ng/ml, upper panel) or mouse IFN-γ (200 U/ml, central panel). In each panel the following stainings are shown from left to right: phospho-Tyr701-STAT1 staining (green), DAPI staining (white) and overlay of the two signals. Activated phospho-STAT1 is indicated by nuclear translocation. Of note, minor background signal in the cytoplasma was observed in some control treated cells. A representative experiment of three replicates is shown. The scale bar represents 50 μm.

*M*. *musculus* NIH/3T3 cells treated with the homologous mouse IFN-γ showed nuclear-localised and phosphorylated Tyr701-STAT1 (Fig. 3). No signal was detected in either untreated NIH/3T3 cells or cells treated with rec*Mg*IFN-γ. As expected from the low IFN-γ sequence similarity between murine and vole species, following treatment of the *M*. *glareolus* kidney cell line BVK168 with mouse IFN-γ we observed no nuclear signal, like in untreated cells (Fig. 3). In contrast, a clear co-localization of phosphorylated Tyr701-STAT1 and cell nuclei was detected in BVK168 cells following rec*Mg*IFN-γ treatment. Furthermore, rec*Mg*IFN-γ but not mouse IFN-γ was active on a *M*. *arvalis* kidney cell line (FMN-R). Moreover, in a cell line (AAL-R) from *A*. *agrarius* mouse IFN-γ activated phosphorylation and nuclear translocation of STAT1 in this related murine species, confirming our predictions from sequence analysis.

Since full transcriptional activity of STAT1 also requires Ser727 being phosphorylated^23^, we assessed its status by an anti-mouse phospho-Ser727-STAT1 antibody in NIH/3T3, BVK168 and AAL-R cell lines. The IFA data mirrored the results of Tyr701 and confirmed Ser727 of STAT1 being also phosphorylated (Supplementary Fig. S2). Together these results provided first evidence of species-specific rec*Mg*IFN-γ cellular activity. These experiments also showed the usefulness of selected reagents (see Supplementary Table S2) for this purpose, i.e. phosphorylation-specific monoclonal antibodies for STAT1, and proved activity of the mouse IFN-γ on a striped field mouse cell line.

### rec*Mg*IFN-γ induces upregulation of an IFN-γ inducible gene

**Figure 4:**
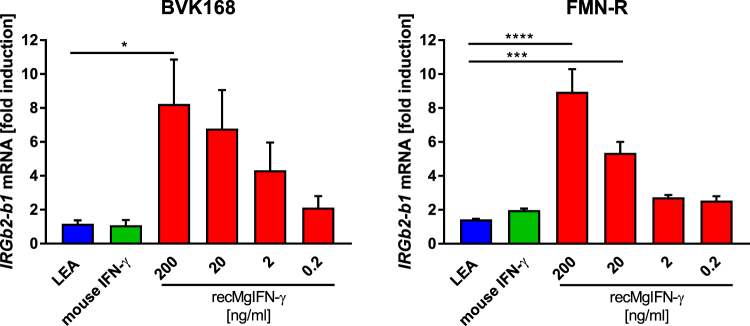
Dose-dependent induction of the expression of the target gene *Irgb2-b1* by rec*Mg*IFN-γ in BVK168 and FMN-R cells. Cells were treated for 24 h with either the negative control protein LEA (200 ng/ml), mouse IFN-γ (200 ng/ml) or rec*Mg*IFN-γ (10-fold dilution from 200 to 0.2 ng/ml). The reported mRNA fold induction is relative to untreated cells (set as 1; not shown) and normalised to *Ywhaz* for reference. Mean ± SEM (n = 3 experiments, each with 2 replicates) are shown. Statistical analysis: One-way Anova followed by Dunnett’s multiple comparisons test (all against the LEA control protein). BVK168 (F(5,12) = 3.75, p = 0.0282) *p = 0.0322, FMN-R (F(5,12) = 21.34, p < 0.0001) ****p = 0.0001, ***p = 0.0004. Note that only statistically significant (p ≤ 0.05) differences were depicted in the figure.

IFN-γ pathway activation leads to STAT1-mediated expression of numerous target genes, such as the *IRG* gene family in rodents^43^. Due to our interest in this innate immune response gene family during infection with the protozoan parasite *T*. *gondii*^44^ we chose to measure the increase of mRNA of one of its members, *Irgb2-b1*, by real-time quantitative Reverse Transcription PCR (qRT-PCR) as a further proof of rec*Mg*IFN-γ activity. The test was performed on BVK168 and FMN-R cells. We compared *Irgb2-b1* expression in cells treated with either rec*Mg*IFN-γ, murine IFN-γ, a control protein (a *T*. *gondii* protein of similar size called LEA) purified from the same *E*. *coli* cells under identical conditions as rec*Mg*IFN-γ, or left untreated. As reference gene for normalization we chose the tyrosine 3-monooxygenase/tryptophan 5-monooxygenase activation protein zeta gene (*Ywhaz)*, which was previously shown to be a reliable gene for this purpose in the closely related field vole *M*. *agrestis*^45^. Following rec*Mg*IFN-γ treatment a dose-dependent relative increase in *Irgb2-b1* mRNA levels in both vole cell lines was observed, which reached 8-fold when 200 ng/ml protein was used, compared to untreated cells or cells treated with the control protein LEA (Fig. 4). The latter confirmed a rec*Mg*IFN-γ-specific effect on this target gene rather than a non-specific cellular stimulation by bacterial contaminants. Taken together, these data demonstrated that rec*Mg*IFN-γ was capable of activating IFN-γ-dependent cellular responses in two cell lines of the two related vole species. However, in the same assay mouse IFN-γ was not active, corroborating the necessity for vole-specific IFN-γ.

### rec*Mg*IFN-γ limits replication of vesicular stomatitis virus in bank vole cells

**Figure 5:**
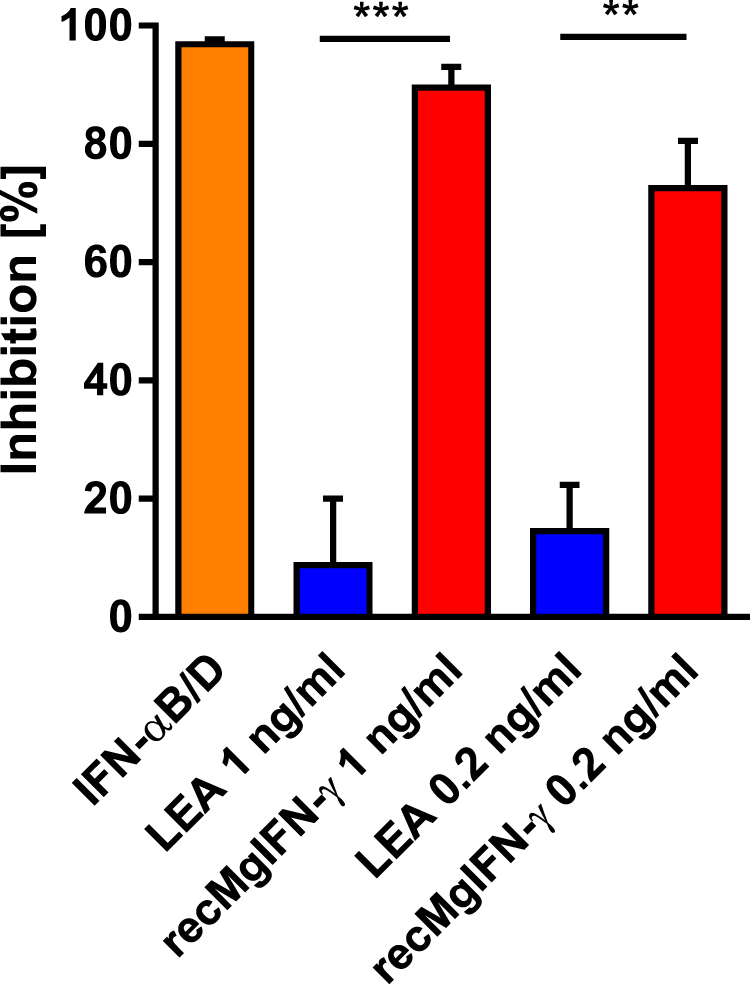
rec*Mg*IFN-γ limits replication of vesicular stomatitis virus in BVK168 cells. Cells infected with the VSV*ΔG(FLuc) strain expressing firefly luciferase were treated with either IFN-αB/D (5 ng/ml) as a known positive effector; with the negative control protein LEA; two concentrations of rec*Mg*IFN-γ (1 and 0.2 ng/ml), or left untreated as control (ctrl). The bioluminescence signal represents viral replication which is reported as percentage relative to untreated cells. Shown are mean ± SEM (n = 3 experiments, each with 2 replicates). Statistical analysis: One-way Anova (F(4, 10) = 37,24, p < 0.0001) followed by Sidak’s multiple comparisons test, **p = 0.0003, ***p < 0.0001.

Testing for antiviral activity is a standard procedure for reporting biological activity of IFN-γ. BVK168 cells were previously shown to be permissive for a number of viruses including vesicular stomatitis virus (VSV)^46^ known to be highly susceptible to IFN-γ treatment in murine cells^47,48^. We used a VSV replicon expressing luciferase (VSV*ΔG(FLuc)) as an easy to follow reporter via bioluminescence readout for measuring the IFN-γ effect on virus replication^49^. As shown in Fig. 5 we observed a drastic reduction of virus replication in VSV-infected BVK168 cells treated with 1 ng/ml rec*Mg*IFN-γ and to a lesser extent with a lower concentration (0.2 ng/ml). The decrease in viral replication was comparable to that of virus-infected cells treated with a human hybrid type I interferon, IFN-αB/D, shown previously to inhibit virus replication^50^. No obvious antiviral effect was seen in cells treated with the control protein LEA. These results demonstrate that rec*Mg*IFN-γ possesses potent antiviral activity using this host-virus system. rec*Mg*IFN-γ antiviral potency was also tested on BVK168 cells infected with two other viruses, cowpox virus (CXPV) Brighton Red strain and murine gammaherpesvirus 68 (MHV68), but led to inconsistent inhibition results (preliminary data). This variability might be due to virus-specific defense mechanisms against host immune responses and/or to different cell type-specific responsiveness already documented in these families of viruses^51–53^.

### Establishment of a bank vole reporter cell line responsive to rec*Mg*IFN-γ

**Figure 6:**
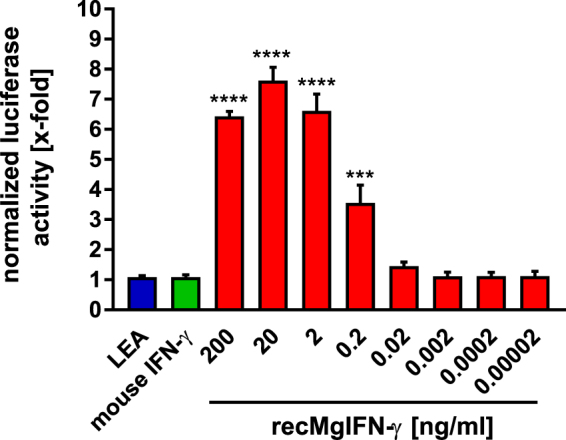
Dose-dependent firefly luciferase activity after recMgIFN-γ induction in a bank vole reporter cell line. Bioluminescence assay on the reporter cell line BVK-LucA expressing the firefly luciferase gene under the control of a GAS promoter element. Cells were either left untreated (ctrl, not shown) or treated with the control protein LEA (200 ng/ml); mouse IFN-γ (200 ng/ml) or recMgIFN-γ (200 to 0.00002 ng/ml). The reported bioluminescence values are relative to untreated cells. Mean ± SEM (n = 4 experiments, each with 3 replicates). Statistical analysis: One-way Anova (F(9, 30) = 76,16, p =  < 0.0001) followed by Dunnett’s multiple comparisons test (all against the LEA control protein). ***p = 0.0002, ****p ≤ 0.0001, relative to the control protein LEA. Note that only statistically significant (p ≤ 0.05) differences were depicted in the figure.

An IFN-γ-responsive, sensitive reporter cell line would be advantageous for different purposes, like studying the influence of pathogens on JAK-STAT signalling^54–56^. Moreover, it would provide an easy test system for functional activity of rec*Mg*IFN-γ. To this end we established a BVK168 reporter cell line (named BVK-LucA) containing a stably integrated plasmid expressing firefly luciferase under the control of a minimal promoter with GAS sites (see Methods for details). As illustrated in Fig. 6, titration of rec*Mg*IFN-γ produced a sigmoidal dose-response in bioluminescence (6 to 8-fold) relative to either untreated, LEA or mouse IFN-γ-treated cells, which is typical for bio-indicator lines^57^. Within the linear range of the assay (between 20 and 0.02 ng/ml) rec*Mg*IFN-γ showed a dose-dependent decrease in signal strength, validating the BVK-LucA cell line as a useful tool to standardise rec*Mg*IFN-γ batches over a 100-fold concentration range. The non-linear dose-response with higher cytokine concentration (200 ng/ml) might be related to the recycling kinetics of the receptor subunit IFN-γR1, as reviewed before^21^, or by IFN-γR1 expression inhibition via an autocrine-mediated loop following persistent activation as already observed for type I interferon^58^.

The observed maximal-fold induction of firefly luciferase in BVK-LucA cells is well in the range of values from other stable GAS-dependent luciferase-expressing cell lines reported in the literature^56,59^. However, gene-specific differences in sequence, length and spacing of the palindromic GAS sequences have been shown to contribute to its affinity for STAT1^60^. Thus, future analyses of vole genomes with regard to GAS elements of known, well-defined IFN-γ-regulated genes might lead to even more sensitive vole reporter cell lines.

## Conclusions

Voles - of which *M*. *glareolus* and *M*. *arvalis* have been proven to be responsive to our recombinant IFN-γ - are epidemiologically important hosts for many pathogens and have been predicted to be reservoirs for one fourth of newly identified zoonoses^3^. For example, natural zoonotic reservoirs of the apicomplexan parasite *Babesia microti*, which can cause malaria-like infections in humans^61^, are various species of voles^13^. The influence of IFN-γ on *B*. *microti* infection has so far only been studied in inbred mouse strains after adaptation of the parasite strains to this unnatural host, and reported experiments eventually led to contradictory results^62,63^.

Voles are also a natural reservoir for the murid herpesvirus 4. Isolates of it (MHV-68) serve as models for human gammaherpesviruses^64^ and IFN-γ is known to control gene expression during MHV-68 latency *in vivo* in mice^65^. However, previous studies have pointed out various differences in pathogenesis between lab mice and natural hosts like *A*. *sylvaticus* and *M*. *glareolus*^66,67^. The provision of unlimited amounts of active recombinant vole IFN-γ reported here will allow the verification of its role in these and other vole-pathogen interactions where in the past house mice had to serve as surrogate and presumably sub-optimal hosts.

## Methods

### Reagents

Mouse IFN-γ (20 µg; Peprotech) was reconstituted in 1 ml sterile water supplemented with 0.1% bovine serum albumin (BSA) and stored in aliquots at −20 °C (100 U = 20 ng). Mouse anti-6His-tag monoclonal antibody used for Western Blotting was from Linaris GmbH (Dossenheim, Germany) and used at a dilution of 0.5 µg/ml. For the immunofluorescent analysis a rabbit anti-phospho-STAT1 (Tyr701) monoclonal antibody (dilution 1:50) or rabbit polyclonal anti-phospho-STAT1 (Ser727) antibody (dilution 1:100; both Cell Signaling Technology) and an AlexaFluor 488-conjugated goat anti-rabbit secondary antibody (dilution 1:1,000; Abcam) were used.

### Culture of cell lines

The following cell lines were used in this study: *Mus musculus* embryonic fibroblast cell line NIH/3T3, *Myodes glareolus* kidney cell line BVK168^46^, *Microtus arvalis* kidney cell line FMN-R and *Apodemus agrarius* lung cell line AAL-R (both Collection of Cell Lines in Veterinary Medicine (CCLV), Friedrich-Loeffler-Institut; Lenk, Reiche, Binder, Riebe and Ulrich, unpublished). All cell lines were maintained in Dulbecco’s modified Eagle’s medium (DMEM) with high glucose, stable glutamine and supplemented pyruvate (DMEM GlutaMAX, ThermoFischer Scientific), supplemented with 10% fetal bovine serum and antibiotics. Cells were passaged twice a week; BVK-168 and FMN-R cells were dissociated with StemPro Accutase (ThermoFischer Scientific), whereas trypsin/EDTA was used for AAL-R and NIH-3T3 cells.

### Expression, purification and characterization of *Mg*IFN-γ

The cDNA sequence of bank vole IFN-γ (GenBank HQ650825) was ordered codon-optimised for *E. coli* from GeneArt (ThermoFischer Scientific) and inserted with a C-terminal 6His-Tag-encoding sequence into a tetracycline-regulated expression vector (pASG-IBA33; IBA Göttingen, Germany) according to the supplier’s instructions. *E*. *coli* OmniMax 2T1R cells (Invitrogen) transformed with the resulting plasmid, named pASG33-*Mg*IFN-γ, were used for expression. Cultures were grown in 2xYT medium at 37 °C to an OD_600_ of 1 before expression was induced by adding 200 ng/ml doxycycline and incubated further for 3 h at 37 °C on an orbital shaker (300 rpm). Cells were lysed by ultrasonication in 25 mM Tris pH 7.5, 500 mM NaCl, 10 mM imidazole, supplemented with EDTA-free cOmplete Protease Inhibitor Cocktail (Roche Diagnostics). The cleared lysate was subjected to immobilised metal affinity chromatography (IMAC) on a Pierce HisPur Chromatography 1 ml cartridge (ThermoFischer Scientific), run on an Äkta Purifier FPLC system (GE Healthcare). Washing of and elution from the column were performed with the same buffer except that 50 and 500 mM imidazole, respectively, was added. Eluates were then subjected to gel filtration chromatography on a 5 ml Sephadex G25 column (GE Healthcare) for buffer exchange to PBS. The resulting protein solution was supplemented with 0.1% BSA and antibiotics and stored in aliquots at −80 °C.

SDS-PAGE and Western blotting was done on a 13% gel according to standard procedures, as described previously^68^. The prestained protein ladder PageRuler 10–180 kDa (ThermoFisher Scientific) was used.

Analytical gel filtration chromatography was performed using the FPLC system and 200 µl of purified rec*Mg*IFN-γ in PBS at room temperature on a calibrated Superdex 75 10/300 GL column (flow rate of 0.75 ml/min) as described previously^69^. Protein extinction was recorded at 230 nm and 260 nm.

Plasmid pQE90S-LEA contains the coding sequence for *T*. *gondii* protein TGME49_076870 of 171 amino acids with a C-terminal 6His-tag and was purchased from GenExpress GmbH (Berlin, Germany). It was transformed into *E*. *coli* OmniMax 2T1R cells, and expression was done as described above, except that for induction isopropyl-β-D-thiogalactopyranosid was added (final concentration 1 mM). Purification, desalting and storage of the recombinant LEA protein were done exactly as described for rec*Mg*IFN-γ.

### Immunofluorescence Assay

BVK168, FMN-R, NIH/3T3 and AAL-R cells were grown on coverslips in 24-well plates and treated for 1 h with either mouse IFN-γ (200 U/ml) or rec*Mg*IFN-γ (200 ng/ml) or left untreated. Cells were then fixed with ice-cold methanol for 20 min, blocked for 1 h in 3% BSA solution, and stained with antibodies (see Reagents section). DNA was stained with 4′,6-Diamidine-2′-phenylindole dihydrochloride (1 ng/ml; Sigma-Aldrich). Coverslips were mounted on microscope slides with Fluoromount Aqueous Mounting Medium (Sigma-Aldrich). Samples were visualised using a Zeiss Axio Imager Z1/Apotome microscope (Zeiss). Images were acquired with a Zeiss AxioCam MRm camera using Zen 2012 Blue edition software and processed using equal linear adjustments for all samples. Image analysis for co-localizations were done using ImageJ 1.49 u and plugin “Blend Images” (http://imagej.nih.gov/ij/plugins/index.html).

### Real-Time PCR

BVK168 and FMN-R cells were seeded into 24-well plates and at 70% confluency treated for 24 h with serial dilutions of rec*Mg*IFN-γ, mouse IFN-γ, LEA or left untreated. Then medium was aspirated and cells were washed once with PBS. Total RNA was extracted with the RNeasy Plus RNA extraction kit after cell lysis with 350 µl RLT Plus buffer supplemented with β-mercaptoethanol (Qiagen) and reverse transcribed to cDNA with the PrimeScript RT-PCR Kit (Takara). PCR primers to amplify *Irgb2-b1* (B2–3′-Fw 5′-AAAGAAGAGCTTYTCACAG-3′ and B1-5′-Rev 5′-TCAGAGAGGATTTTRCTTTC-3′) were designed on available *Microtus ochrogaster* and *M*. *musculus* sequences (see Supplementary Methods). PCR primers to amplify the reference gene tyrosine 3-monooxygenase/tryptophan 5-monooxygenase activation protein zeta gene (*Ywhaz*) were based on the homologous *M*. *agrestis* sequence reported previously^45^ (primer L-Ywhaz 5′-GATGAAGCCATTGCTGAACTTGA-3′ and R-Ywhaz 5′-GCCGGTTAATTTTCCCCTCCT-3′). The amplicon extends over intron 4 with 1764 bp in *M*. *ochrogaster*, whereas the mRNA-derived product is 155 bp. These primers also bind to the respective *M*. *musculus* sequence (see Supplementary Methods). Real-Time PCR was performed with the Maxima SYBR Green/ROX kit (ThermoFisher Scientific) on an Applied Biosystem ABI 7500 Real-Time PCR system. All reactions were run in duplicate with a no-template control to check for contaminations. Each tube contained 1 μl of cDNA template (equivalent to 5 ng), 12.5 μl SYBR Green mix solution, 0.75 μl primer (75 μM each) and 10 μl H_2_O for a total reaction volume of 25 μl. The PCR conditions were 2 min at 50 °C, 10 min at 95 °C and 40 cycles of each 95° for 15 s and 60 °C for 1 min. Melting curve analysis was performed from 60° to 95 °C at 1% ramp rate, with each step lasting 30 s to confirm presence of a single product and absence of primer dimers.

### Establishment of the reporter cell line and luciferase assay

To allow selection for stable clones the blasticidin resistance selection marker (BsdR) gene was inserted into plasmid pGAS-Luc (Stratagene), which consists of a synthetic promoter that contains four direct repeats of a human-derived transcription recognition sequence for STAT1 (AGTTTCATATTACTCTAAATC)_4_ and a GAS followed by the firefly luciferase gene. The BsdR coding unit encompassed the SV40 promoter and origin, the Blasticidin resistance gene and the SV40 early polyadenylation site. It was amplified from pcDNA6-TR (Invitrogen) using primers Bsd-fwd 5′-TCATGATAATAATGGTTTCTTAGACCAGACATGATAAGATACATTG-3′ and Bsd-rev 5′-ATTTCCCCGAAAAGTGCCACCTGACGAATGTGTGTCAGTTAGGG-3′. Cloning of the PCR product into ZraI-linearised pGAS-Luc was performed via the Seamless Ligation Cloning Extract (SLiCE) method as described in^70^ using *E*. *coli* strain JM109(DE3) (Promega) containing plasmid pKD46^71^ (Coli Genetic Stock Center #64672). The resulting plasmid, pGAS-Luc-Bsd, was sequenced over the insertion sites.

BVK168 cells were transfected with the plasmid pGAS-Luc-Bsd via the 25 kDa linear cationic polymer polyethylenimine (PEI; Polysciences) in a molar ratio DNA:PEI of 1:500. Using Blasticidin selection (20 μg/ml, InvivoGen) a stably transfected BVK168 cell line was obtained and named BVK-LucA.

For the luciferase assay BVK-LucA cells were seeded into a 48-well plate. After 24 h either serial dilutions of rec*Mg*IFN-γ or mouse IFN-γ and LEA or DMEM as controls were added and incubation was continued for 330 min at 37 °C as described previously^59^. The supernatant was removed, cells were washed once with PBS and 65 μl of lysis reagent was added to each well. The lysates were assayed using the Dual-Luciferase Reporter Assay System (Promega). 20 µl of the cell lysate was mixed with 100 µl of reconstituted luciferase assay reagent in a 96 well plate and the emitted light was measured in a Tristar LB 941 luminometer (Berthold).

### Antiviral activity assay

For the antiviral activity assays BVK168 cells were seeded into 24-well plates and 6 h later treated with either rec*Mg*IFN-γ (1:200 and 1:1,000 dilutions, equivalent to 1 ng/ml and 0.2 ng/ml, respectively), with the control protein LEA (in concentration analogous to rec*Mg*IFN-γ) or left untreated for 16 h before infection. In addition, recombinant human IFN-αB/D known to be active on *M*. *glareolus* cell line was used as positive control (5 ng/ml)^50^. Next, the medium was aspirated and cells were inoculated with 0.5 MOI of VSV*ΔG(FLuc), a VSV glycoprotein-pseudotyped, propagation-incompetent VSV replicon, expressing firefly luciferase^49^. At 24 h post inoculation cells were lysed in 80 μl passive lysis buffer per well and luciferase activity determined in duplicates in a luminometer via the Dual-Luciferase Reporter Assay System (Promega).

### Software used for sequence and statistical analyses

Sequences were searched by BLAST using the respective *M*. *musculus* protein sequences as query and downloaded from NCBI as detailed in SI Methods. Protein sequences were assembled from translated contigs, based on the respective BLAST results and comparison with those of the GeneWise server (https://www.ebi.ac.uk/Tools/psa/genewise). Multiple sequence alignments were performed using MUSCLE 3.8.31 and visualised with JalView 2.10.1^72^. The taxonomic species trees in Fig. 1 and Supplementary Fig. S1 (with species names as query) were obtained from https://www.ncbi.nlm.nih.gov/Taxonomy/CommonTree/wwwcmt.cgi, visualised using the Evolview V2 server^73^ or FigTree 1.4.3 (http://tree.bio.ed.ac.uk/software/figtree/) and modified with AffinityDesigner 1.5.3 software (Serif Ltd, UK).

Statistical analyses were performed using Prism 7 (GraphPad). Data were tested for normal distribution with the D’Agostino and Pearson normality test.

### Data availability

All data generated or analysed during this study are included in the published article and its Supplementary Information files. Biological materials described herein are available upon request.

## Electronic supplementary material

Supplementary Information

## References

[1] Karesh, W. B. *et al*. Ecology of zoonoses: natural and unnatural histories. *Lancet* **380**, 1936–1945 (2012). 10.1016/S0140-6736(12)61678-X PMC7138068 23200502

[2] Meerburg BG, Singleton GR, Kijlstra A (2009). Rodent-borne diseases and their risks for public health. Crit. Rev. Microbiol..

[3] Han BA, Schmidt JP, Bowden SE, Drake JM (2015). Rodent reservoirs of future zoonotic diseases. Proc. Natl. Acad. Sci. USA.

[4] Ermonval, M., Baychelier, F. & Tordo, N. What do we know about how hantaviruses interact with their different hosts? *Viruses* **8** (2016). 10.3390/v8080223 PMC4997585 27529272

[5] Achazi K (2011). Rodents as sentinels for the prevalence of tick-borne encephalitis virus. Vector Borne Zoonotic Dis..

[6] Hoffmann D (2015). Out of the reservoir: phenotypic and genotypic characterization of a novel cowpox virus isolated from a common vole. J. Virol..

[7] Kinnunen PM, Palva A, Vaheri A, Vapalahti O (2013). Epidemiology and host spectrum of Borna disease virus infections. J. Gen. Virol..

[8] Perec-Matysiak A, Bunkowska-Gawlik K, Zalesny G, Hildebrand J (2015). Small rodents as reservoirs of Cryptosporidium spp. and Giardia spp. in south-western Poland. Ann. Agric. Environ. Med..

[9] Schmidt S (2014). Multiple infections of rodents with zoonotic pathogens in Austria. Vector Borne Zoonotic Dis..

[10] Tadin A (2016). Molecular survey of zoonotic agents in rodents and other small mammals in Croatia. Am. J. Trop. Med. Hyg..

[11] Kilonzo C (2013). Fecal shedding of zoonotic food-borne pathogens by wild rodents in a major agricultural region of the central California coast. Appl. Environ. Microbiol..

[12] Obiegala A (2016). Prevalence and genotype allocation of pathogenic leptospira species in small mammals from various habitat types in Germany. PLoS Negl. Trop. Dis..

[13] Yabsley MJ, Shock BC (2013). Natural history of zoonotic Babesia: role of wildlife reservoirs. Int. J. Parasitol. Parasites Wildl..

[14] Ehret, T., Torelli, F., Klotz, C., Pedersen, A. B. & Seeber, F. Translational rodent models for research on parasitic protozoa – a review of confounders and possibilities. *Front*. *Cell*. *Infect*. *Microbiol*. **7**, 238 (2017). 10.3389/fcimb.2017.00238 PMC5461347 28638807

[15] Jackson JA (2015). Immunology in wild nonmodel rodents: an ecological context for studies of health and disease. Parasite Immunol..

[16] Pedersen AB, Babayan SA (2011). Wild immunology. Mol. Ecol..

[17] Mulugeta E (2016). Genomes of Ellobius species provide insight into the evolutionary dynamics of mammalian sex chromosomes. Genome Res..

[18] Bedford NL, Hoekstra HE (2015). Peromyscus mice as a model for studying natural variation. eLife.

[19] Zimmerman LM, Bowden RM, Vogel LA (2014). A vertebrate cytokine primer for eco-immunologists. Funct. Ecol..

[20] Schoenborn JR, Wilson CB (2007). Regulation of interferon-gamma during innate and adaptive immune responses. Adv. Immunol..

[21] de Weerd NA, Nguyen T (2012). The interferons and their receptors|[mdash]|distribution and regulation. Immunol. Cell Biol..

[22] Hu X, Ivashkiv LB (2009). Cross-regulation of signaling pathways by interferon-gamma: implications for immune responses and autoimmune diseases. Immunity.

[23] Wen Z, Zhong Z, Darnell JE (1995). Maximal activation of transcription by Stat1 and Stat3 requires both tyrosine and serine phosphorylation. Cell.

[24] Bekpen C (2005). The interferon-induciblep47 (IRG) GTPases in vertebrates: loss of the cell autonomous resistance mechanism in the human lineage. Genome Biol..

[25] Al-Zeer MA, Al-Younes HM, Braun PR, Zerrahn J, Meyer TF (2009). IFN-gamma-inducible Irga6 mediates host resistance against Chlamydia trachomatis via autophagy. PLoS ONE.

[26] Liesenfeld O (2011). The IFN-gamma-inducible GTPase, Irga6, protects mice against Toxoplasma gondii but not against Plasmodium berghei and some other intracellular pathogens. PLoS ONE.

[27] Novelli F, Casanova JL (2004). The role of IL-12, IL-23 and IFN-gamma in immunity to viruses. Cytokine Growth Factor Rev..

[28] Shtrichman R, Samuel CE (2001). The role of gamma interferon in antimicrobial immunity. Curr. Opin. Microbiol..

[29] Savan R, Ravichandran S, Collins JR, Sakai M, Young HA (2009). Structural conservation of interferon gamma among vertebrates. Cytokine Growth Factor Rev..

[30] Randal M, Kossiakoff AA (2001). The structure and activity of a monomeric interferon-gamma:alpha-chain receptor signaling complex. Structure.

[31] Lundell D (1991). The carboxyl-terminal region of human interferon-gamma is important for biological-activity - mutagenic and NMR analysis. Protein Eng..

[32] Döbeli H, Gentz R, Jucker W, Garotta G (1988). Role of the carboxy-terminal sequence on the biological activity of human immune interferon (IFN-γ). J. Biotec..

[33] Slodowski O, Bohm J, Schone B, Otto B (1991). Carboxy-terminal truncated rhuIFN-gamma with a substitution of Gln133 or Ser132 to leucine leads to higher biological activity than in the wild type. Eur. J. Biochem..

[34] Nacheva G (2003). Human interferon gamma: significance of the C-terminal flexible domain for its biological activity. Arch. Biochem. Biophys..

[35] Arakawa T, Alton NK, Hsu YR (1985). Preparation and characterization of recombinant DNA-derived human interferon-gamma. J. Biol. Chem..

[36] Arakawa T (1990). Reversibility of acid denaturation of recombinant interferon-gamma. Biopolymers.

[37] Kendrick BS (1998). Aggregation of recombinant human interferon gamma: kinetics and structural transitions. J. Pharm. Sci..

[38] Tobler SA, Fernandez EJ (2002). Structural features of interferon-gamma aggregation revealed by hydrogen exchange. Protein Sci..

[39] Sugimura K, Higashi N (1988). A novel outer-membrane-associated protease in Escherichia coli. J. Bacteriol..

[40] Sugimura K, Nishihara T (1988). Purification, characterization, and primary structure of Escherichia coli protease VII with specificity for paired basic residues: identity of protease VII and OmpT. J. Bacteriol..

[41] Gorissen M, de Vrieze E, Flik G, Huising MO (2011). STAT genes display differential evolutionary rates that correlate with their roles in the endocrine and immune system. J. Endocrinol..

[42] Stoltz, M. *et al*. A model system for *in vitro* studies of bank vole borne viruses. *PLoS ONE* **6** (2011). 10.1371/journal.pone.0028992 PMC3241689 22194969

[43] Martens S, Howard J (2006). The interferon-inducible GTPases. Annu. Rev. Cell Dev. Biol..

[44] Lilue J, Müller UB, Steinfeldt T, Howard JC (2013). Reciprocal virulence and resistance polymorphism in the relationship between Toxoplasma gondii and the house mouse. eLife.

[45] Jackson JA (2010). The analysis of immunological profiles in wild animals: a case study on immunodynamics in the field vole, Microtus agrestis. Mol. Ecol..

[46] Essbauer SS, Krautkrämer E, Herzog S, Pfeffer M (2011). A new permanent cell line derived from the bank vole (Myodes glareolus) as cell culture model for zoonotic viruses. Virol. J..

[47] Voigt E, İnankur B, Baltes A, Yin J (2013). A quantitative infection assay for human type I, II, and III interferon antiviral activities. Virol. J..

[48] Chesler DA, Dodard C, Lee GY, Levy DE, Reiss CS (2004). Interferon-gamma-induced inhibition of neuronal vesicular stomatitis virus infection is STAT1 dependent. J. Neurovirol..

[49] Berger Rentsch M, Zimmer G (2011). A vesicular stomatitis virus replicon-based bioassay for the rapid and sensitive determination of multi-species type I interferon. PLoS ONE.

[50] Horisberger MA, de Staritzky K (1987). A recombinant human interferon-alpha B/D hybrid with a broad host-range. J. Gen. Virol..

[51] Liang X, Shin YC, Means RE, Jung JU (2004). Inhibition of interferon-mediated antiviral activity by Murine Gammaherpesvirus 68 latency-associated M2 protein. J. Virol..

[52] Trilling M (2009). Gamma interferon-induced Interferon Regulatory Factor 1-dependent antiviral response inhibits vaccinia virus replication in mouse but not human fibroblasts. J. Virol..

[53] Steed, A., Buch, T., Waisman, A. & Virgin, H. Gamma interferon blocks gammaherpesvirus reactivation from latency in a cell type-specific manner. *J. Virol.* **81**, 6134–6140 (2007). 10.1128/JVI.00108-07 PMC1900319 17360749

[54] Kincaid EZ, Ernst JD (2003). Mycobacterium tuberculosis exerts gene-selective inhibition of transcriptional responses to IFN-gamma without inhibiting STAT1 function. J. Immunol..

[55] Rodriguez JJ, Parisien JP, Horvath CM (2002). Nipah virus V protein evades alpha and gamma interferons by preventing STAT1 and STAT2 activation and nuclear accumulation. J. Virol..

[56] Rosowski EE, Saeij JPJ (2012). Toxoplasma gondii clonal strains all inhibit STAT1 transcriptional activity but polymorphic effectors differentially modulate ifnγ induced gene expression and STAT1 phosphorylation. PLoS ONE.

[57] Grossberg SE, Kawade Y, Kohase M, Yokoyama H, Finter N (2001). The neutralization of interferons by antibody. I. Quantitative and theoretical analyses of the neutralization reaction in different bioassay systems. J. Interferon Cytokine Res..

[58] Dupont SA, Goelz S, Goyal J, Green M (2002). Mechanisms for regulation of cellular responsiveness to human IFN-beta1a. J. Interferon Cytokine Res..

[59] Schäfer H (2007). Biologic activity of guinea pig IFN-γ *in vitro*. J. Interferon Cytokine Res..

[60] Decker T, Kovarik P, Meinke A (1997). GAS elements: A few nucleotides with a major impact on cytokine-induced gene expression. J. Interferon Cytokine Res..

[61] Vannier EG, Diuk-Wasser MA, Ben Mamoun C, Krause PJ (2015). Babesiosis. Infect. Dis. Clin. North Am..

[62] Clawson ML (2002). Cellular immunity, but not gamma interferon, is essential for resolution of Babesia microti infection in BALB/c mice. Infect. Immun..

[63] Igarashi I (1999). Roles of CD4(+) T cells and gamma interferon in protective immunity against Babesia microti infection in mice. Infect. Immun..

[64] Dong S, Forrest JC, Liang X (2017). Murine gammaherpesvirus 68: a small animal model for gammaherpesvirus-associated diseases. Adv. Exp. Med. Biol..

[65] Steed AL (2006). Gamma interferon blocks gammaherpesvirus reactivation from latency. J. Virol..

[66] Francois S (2010). Comparative study of murid gammaherpesvirus 4 infection in mice and in a natural host, bank voles. J. Gen. Virol..

[67] Hughes DJ, Kipar A, Leeming G, Sample JT, Stewart JP (2012). Experimental infection of laboratory-bred bank voles (Myodes glareolus) with murid herpesvirus 4. Arch. Virol..

[68] Vollmer M, Thomsen N, Wiek S, Seeber F (2001). Apicomplexan parasites possess distinct nuclear-encoded, but apicoplast-localized, plant-type ferredoxin-NADP + reductase and ferredoxin. J. Biol. Chem..

[69] Frohnecke N, Klein S, Seeber F (2015). Protein-protein interaction studies provide evidence for electron transfer from ferredoxin to lipoic acid synthase in Toxoplasma gondii. FEBS Lett..

[70] Zhang Y, Werling U, Edelmann W (2012). SLiCE: a novel bacterial cell extract-based DNA cloning method. Nucleic Acids Res..

[71] Datsenko KA, Wanner BL (2000). One-step inactivation of chromosomal genes in Escherichia coli K-12 using PCR products. Proc. Natl. Acad. Sci. USA.

[72] Waterhouse AM, Procter JB, Martin DM, Clamp M, Barton GJ (2009). Jalview Version 2–a multiple sequence alignment editor and analysis workbench. Bioinformatics.

[73] He Z (2016). Evolviewv2: an online visualization and management tool for customized and annotated phylogenetic trees. Nucleic Acids Res..

