# Supplementary material for: Recombinant IFN-γ from the bank vole Myodes glareolus: a novel tool for research on rodent reservoirs of zoonotic pathogens
Source: Sci Rep. 2018 Feb 12;8:2797. doi: 10.1038/s41598-018-21143-0 (PMC5809609; doi:10.1038/s41598-018-21143-0)
Supplement: Supplementary file 1 — Supplementary Information [file 41598_2018_21143_MOESM1_ESM.pdf]

Recombinant IFN-γ from the bank vole *Myodes glareolus*: a novel tool for research on rodent reservoirs of zoonotic pathogens

Francesca Torelli[a], Steffen Zander[a], Heinz Ellerbrok[b], Georg Kochs[c], Rainer G. Ulrich[d], Christian Klotz[a], Frank Seeber[a*]

[a] Department of Mycotic and Parasitic Agents and Mycobacteria, Robert Koch-Institut, Berlin, Germany

[b] Center for Biological Threats and Special Pathogens, Highly Pathogenic Viruses, Robert Koch-Institut, Berlin, Germany

[c] Institute of Virology, Medical Center – University of Freiburg, Faculty of Medicine, University of Freiburg, Germany

[d] Institute of Novel and Emerging Infectious Diseases, Friedrich-Loeffler-Institut, Federal Research Institute for Animal Health, Greifswald-Insel Riems, Germany

[*]Corresponding author: seeberf@rki.de

Supplementary Methods

Related to Fig. 1:

BLAST servers used for vole genomes:

BLAST Microtus agrestis

BLAST Apodemus sylvaticus

BLAST Myodes glareolus

BLAST Ellobius lutescens

Assembly of IFN-γ sequences from draft genomes:

Apodemus sylvaticus: assembled from whole genome shotgun sequences:

Scaffold6812 (https://www.ncbi.nlm.nih.gov/Traces/wgs/LIPJ01/LIPJ01006807)

C77709064 (https://www.ncbi.nlm.nih.gov/Traces/wgs/LIPJ01/LIPJ01502169)

Myodes glareolus: assembled from whole genome shotgun sequences:

scaffold141145 (https://www.ncbi.nlm.nih.gov/Traces/wgs/LIPI01/LIPI01140256)

scaffold60943 (https://www.ncbi.nlm.nih.gov/Traces/wgs/LIPI01/LIPI01060555)

scaffold6355 (https://www.ncbi.nlm.nih.gov/Traces/wgs/LIPI01/LIPI01006333)

Microtus agrestis: assembled from whole genome shotgun sequences:

scaffold3995 (https://www.ncbi.nlm.nih.gov/Traces/wgs/LIQJ01/LIQJ01003986)

Ellobius lutescens: assembled from whole genome shotgun sequence:

scaffold8069 https://www.ncbi.nlm.nih.gov/Traces/wgs/LOEQ01/LOEQ01008069

Other sequences retrieved via the following accession numbers:

Peromyscus maniculatus bairdii XP_006973418.1

Microtus ochrogaster XP_005357970.1

Rattus norvegicus NP_620235.1

Homo sapiens NP_000610.2

Mus musculus BL6 NP_032363.1

Taxonomic tree in Fig. 1A was according to

https://www.ncbi.nlm.nih.gov/Taxonomy/CommonTree/wwwcmt.cgi

Related to Fig. 4:

putative *Irgb2-b1* sequence from *Microtus ochrogaster* for qPCR primer design:

https://www.ncbi.nlm.nih.gov/nuccore/XM_005350187

Microtus ochrogaster tyrosine 3-monooxygenase/tryptophan 5-monooxygenase activation protein, zeta (Ywhaz), transcript variant X1:

https://www.ncbi.nlm.nih.gov/nuccore/913515031

>> its genomic sequence used for primer design:

NW_004949127.1:1460000-1482000 *Microtus ochrogaster* isolate Prairie Vole_2

https://www.ncbi.nlm.nih.gov/nuccore/530286854?report=fasta&from=1460000&to=1482000

>> qPCR primers extend over intron 4, with 1764 bp, whereas mRNA-derived product is 155 bp

Mus musculus tyrosine 3-monooxygenase/tryptophan 5-monooxygenase activation protein, zeta polypeptide (Ywhaz), transcript variant X1:

https://www.ncbi.nlm.nih.gov/nuccore/NM_011740.3

Related to Figure S1:

Data extracted from supplementary data from: Han, B. A., Schmidt, J. P., Bowden, S. E. & Drake, J. M. Rodent reservoirs of future zoonotic diseases. *Proceedings of the National Academy of Sciences* **112,** 7039–7044 (2015) and "Rodent reservoirs of future zoonotic diseases. Dryad Digital Repository. https://doi.org/10.5061/dryad.7fh4q ". Species names were extracted from this table in which reservoirs are indicated as „flag".

Supplementary Fig. S1

Species of voles, *Peromyscus* spp. and *Apodemus* spp. within known or predicted zoonotic rodent reservoir hosts. Analysis is based on supplementary data from Han et al (2015). **A** 43 out of 217 (19.8%) known zoonotic rodent reservoir hosts are either voles, *Peromyscus* spp. or *Apodemus* spp. species, respectively (highlighted in red, blue and green). **B** From 57 predicted new rodent zoonotic reservoir hosts 16 are voles (28%; highlighted in red).

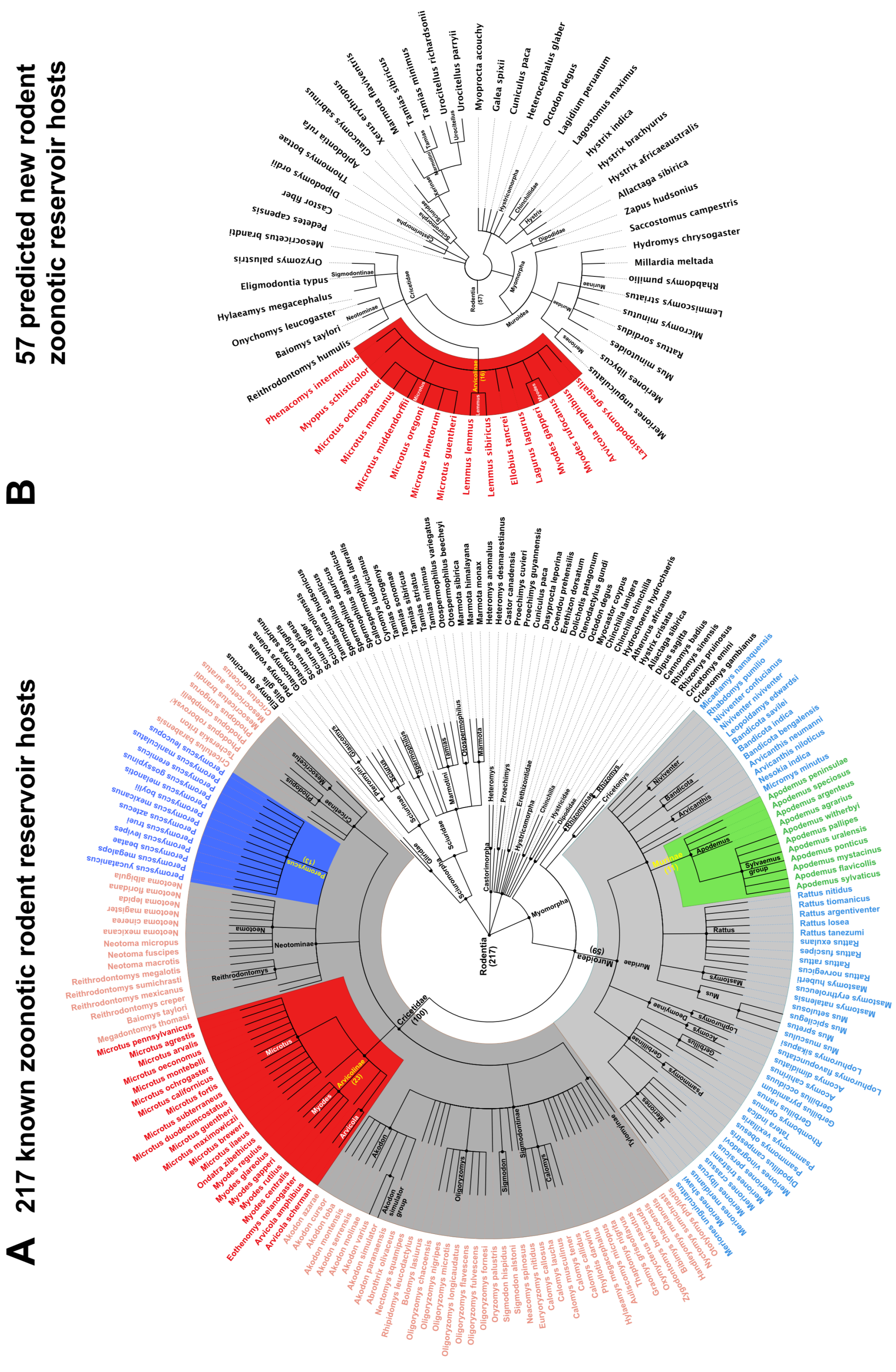

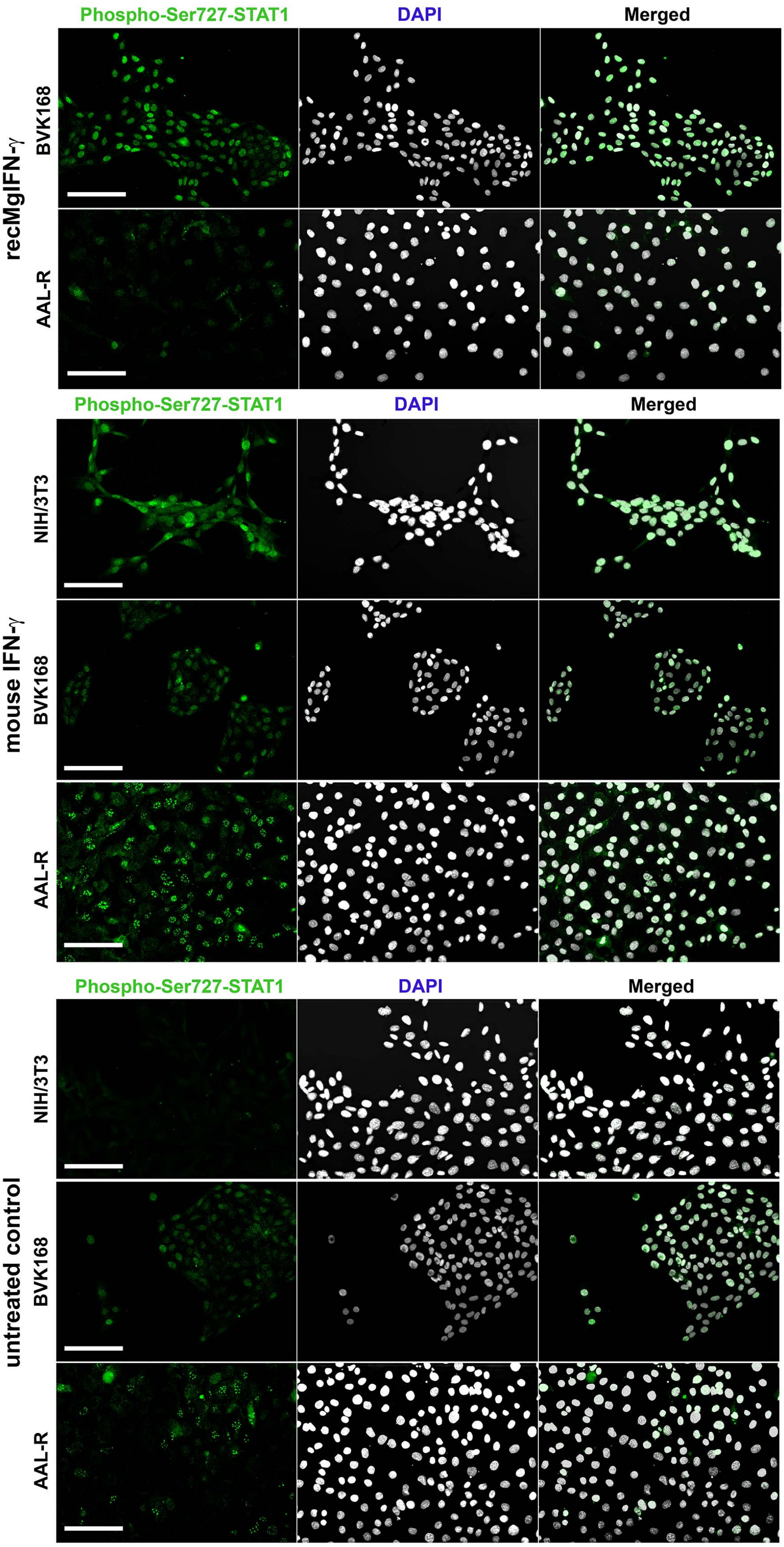

Supplementary Fig. S2

Ser727-STAT1 phosphorylation confirms the activity of recMgIFN-γ on BVK168 cell line and of mouse IFN-γ on AAL-R cell line.

Cells were either stimulated for 1h with rec*Mg*IFN-γ (200 ng/ml, upper panel), with mouse IFN-γ (200 U/ml, central panel) or left untreated as control (lower panel). In each panel the following staining is shown from left to right: phospho-Ser727-STAT1 staining (green), DAPI staining (white) and overlay of the two signals.

A representative experiment of two replicates is shown. The scale bar represents 100 μm.

Supplementary Table S1: Commercial reagents

Immune Reagents	Reagent Species	Cross-reactivity	Reference
recIFN-αB/D	*Homo sapiens*	*M. glareolus* BVK168 cell line	Horisberger, M. A. & de Staritzky, K. *J Gen Virol* **68 (Pt 3)**, 945-948 (1987)
IFN-γ	*M. musculus*	*A. agrarius* AAL-R cell line	Peprotech Cat. n° 315-05
**Primary antibodies**			
mAb anti-Phospho-Stat1 (Tyr701)	rabbit anti-mouse	*M. glareolus* BVK168<br>*M. arvalis* FMN-R<br>*A. agrarius* AAL-R cell lines	Cell Signaling Technology Cat. n° 9167
mAb anti-Phospho-Stat1 (Ser727)	rabbit anti-mouse	*M. glareolus* BVK168<br>*A. agrarius* AAL-R cell lines	Cell Signaling Technology Cat. n° 8826
